# Collectanea Medica, Consisting of Anecdotes, Facts, Extracts, Illustrations, Queries, Suggestions, &c.

**Published:** 1817-12

**Authors:** 


### COLLECTANEA MEDICA,

CONSISTING OF

### ANECDOTES, FACTS, EXTRACTS, ILLUSTRATIONS, QUERIES, SUGGESTIONS, &c.

RELATING TO THE

***History or the Art of Medicine, and the Auxiliary Sciences.***

Quicquid agunt medici, nostri farrago libelli.

*History of a Case of Rupture of the Brain and its Membranes, arising from the Accumulation of Fluid, in a Case of Hydrocephalus Internus.* By JOHN BARON, M.D. Physician to the Infirmary at Glocester. (From the Medico-Chirurg. Trans.)

SARAH BARGUM was born on the 18th of September, 1815. At the time of birth the head was observed to be remarkably large. She was brought to me at Glocester, on the 11th of the following December. At that time the circumference of the head was twenty-eight inches. In the course of another week it increased about an inch. The bones were separated from each other to the greatest possible degree, and all the divisions of the fœtal skull were very observable. The circumference did not further enlarge, but a swelling began on the top of the head over the posterior fontanelle, which, in the space of another week, acquired the magnitude of a goose's egg. At this period of the disease, the mother, on going one morning to take up the child, was very much surprised to find that the swelling had become much smaller, and perfectly soft. She observed likewise a constant dribbling of water from the urinary passage, and that the bed was soaked with the discharge. It continued incessantly for three days and three nights. By this time the swelling had entirely disappeared, the head was considerably smaller, and the integuments, which before were very much distended, now fell in large wrinkles over the child's forehead, so as actually to cover the eyes.

An increased flow from the urinary organs continued for nearly two months. When it became smaller in quantity, the head began again rapidly to enlarge, the swelling on the top re appeared, and acquired a much greater bulk than before, it having on this occasion extended extended itself over the head and part of the face. On the 7th of March, the tumour had arrived at its greatest size. On that day a watery discharge, tinged with blood, was seen to ooze from the nostrils and mouth. It continued without ceasing till the 10th, when the swelling on the top of the head had vanished, and the head itself was much smaller.

The fluid never again accumulated in the sac on the outside, nor did the head ever gain its former magnitude, because the discharge from the nostrils was kept up, with slight intermission, till the time of its death on the 8th of this month. When, by any accident, the oozing from the nostrils ceased, the parents affirm that there was a corresponding increase in the urinary discharge. When the head was held forward, the fluid ran freely from the nostrils.

The child continued to eat well to the last. The evacuations from the bowels are reported to have been natural, but its powers of assimilation appear to have been destroyed, as she did not seem to have increased in bulk from the time I first saw it. She was sensible to external impressions, but the parents were not in that rank of life to make many observations, either on the nature or effect of such impressions. She never appears to have made the slightest attempt to articulate.

As she lived in the forest of Dean, I had it not in my power to examine her so frequently as I could have wished. But the foregoing facts were perfectly established, by what I saw myself, and by the testimony of a friend* who lives in the neighbourhood, and had an opportunity of ascertaining their truth. The second filling of the head, after it had been emptied by the great discharge from the urinary organs, seems to have been accomplished in little more than a fortnight. For though the first swelling disappeared in three days, the secretion from the kidnies appears to have been sufficiently active to prevent a second accumulation, till towards the end of February, when the swelling was again observed. As has been already noticed, it gained its greatest size on the 7th of March, and on the 10th it was no more visible.

On the 10th of this month I went to examine the child. Being obliged to perform the dissection in presence of the parents, it was less minute in some respects than I could have wished.

The circumference of the head was about 20 inches, being less by nine inches than it was before the swelling on the top first appeared.

I made a crucial incision, and dissected back the integuments. On uncovering that part formerly occupied by the swelling, I saw immediately what accounted for its origin, and explained many of the phenomena. A little to the right side of the falx the dura mater was ruptured, as was demonstrated by a well-defined circular opening nearly one inch in diameter, which communicated directly with what was the external tumour and the interior of the brain. Through

* J. Pyrke, Esq. of Little Dean.

this opening I evacuated between three and four pints of fluid, which was contained in a bag formed by both hemispheres of the brain. The expansion of the brain was so great, that round the margin of the opening of the dura mater, it did not equal the thickness of a shilling; and under the opening it had entirely disappeared, proving that it had given way when the dura mater yielded, and allowed the fluid from the internal cavity to escape into the outward swelling.

The cerebellum was entire, and the organs of the different nerves seemed unimpaired. I could not well examine the æthmoid bone, but I easily passed a probe throught it into the nose.

The inferences deducible from this case are so obvious, that I forbear to mention them. I am not aware that any of a similar kind has ever been recorded. For that reason it appears to me not unworthy of a place in the Memoirs of this Society.

The expansion of the brain, of its membranes and of the cranium, seems to have gone on till the parts would stretch no longer. Then the rupture took place which caused the first swelling, and established a free and large communication between it and the interior of the brain. This fact is demonstrated by the rapid and remarkable absorption of the fluid in the tumour and in the head, so that the first entirely disappeared, and the latter became so much diminished in size, that the integuments hung in large folds, as already described. We have more convincing illustration of the same fact in the second appearance of the swelling. It was not circumscribed as at first, but extended over the head and face.

I have seen one case similar to that recorded by Mr. Earle in the last volume of the Transactions. The tumour was in the same situation, and had been twice punctured. The event of the disease has not yet been ascertained.

*Glocester; Feb.* 28, 1817.

---

### *On the internal and external Use of the Nitro-Muriatic Acid, in the Cure of Diseases.* By H. Scott, M.D. (From the same.)

[In the first article of our Analysis, we have given our opinion on the remedy recommended below. As, however, the subject is more particularly concentrated by the author himself in the following paper, we have thought it right to select the most important part for this division of our labours.]

I left India in very bad health, and long remained ill in this country. During this period I often reflected on my experience with the acids, and resolved, as soon as an opportunity should occur, to return to the use of the nitro-muriatic acid, and to satisfy my mind on the subject from further observation. Above a year ago I came to London, and had an opportunity of getting the nitro-muriatic acid employed to a considerable extent. At first, I mixed three parts of nitrous acid with one of muriatic, in order to imitate the acid I had used in India. Of late, however, I have employed the acids in equal weights, and I give a preference to this proportion; tion; but there may be others still more efficacious which time will discover.

Although I had reason in India to be satisfied with the general effects derived from this acid, yet considerable inconvenience attended its internal use. If it were not given with great caution, it injured the enamel of the teeth, though not their roots, as mercury does. Even in small quantities, it disagreed with some stomachs, and few individuals indeed could continue it sufficiently long, or in sufficient quantity, to remove from the system the symptoms of secondary syphilis. These difficulties made me anxious to ascertain whether or not the acid, externally applied, would produce good effects. I accordingly employed it in this manner in various cases, and I immersed my own body up to the chin in a bath of this acid, sufficiently diluted with water. I fortunately have preserved the memoranda that I made at the time, of which the following is a copy.

"*Bombay;* 27th *April,* 1798.—I bathed to-day in an acid bath, which was merely acidulous. It covered the whole body below the head. I staid in it for half an hour, and it was nearly of the temperature of the body. I feel no particular effect from this bath: it is fully as pleasant as water, and cleanses the skin like a soap.

"*April* 28. I bathed again to-day, keeping the bath at the same temperature, or making it rather higher, of the same strength with regard to acid. I staid in it half an hour. I still feel no material effect from the bath. Pulse after bathing 76°. The only apparent action of the acid is on such animal matters as are unprotected by life, with which it forms an acid soap. Would it not deprive feathers of their oily smell?

"29th. Bathed again, and continued, as before, for half an hour in the bath. To-day the bath was hardly so hot as the body. About half an hour after bathing yesterday, I became sensible of an odd sensation about my gums, my jaws, and my teeth.

"30th. Bathed again, and stayed half an hour in the bath. It was rather warmer than my body. Since yesterday I have been sensible of some uneasiness in my throat on swallowing. I feel a disposition to salivation at times, but I am otherwise well. My gums, both above and below, are somewhat reddened. I was for some time disposed to ascribe these effects to imagination, but they have continued all this day, and leave me no doubt of their reality. I am in good health.

"*May* 1st. Since yesterday I have felt some pain in my throat, especially on swallowing. This pain seems to follow the course of the œsophagus. During the whole of this forenoon I had a sense of *burning* over the roof of my mouth and down the gullet. This sensation is like what arises from having chewed an acrid vegetable substance, and is so unpleasant, that, unless it leave me by to-morrow, I shall bathe no more. To-day I bathed as usual, staying in the bath for half an hour. It has been to-day, and, in general, so acid, as to make my skin smart a good deal in many places.

"2d. My mouth, &c. though not in the least ulcerated, is somewhat what pained. I am sufficiently convinced of the great power of this bath, and shall bathe no more. My digestion is improved, and I feel that my liver, unclogged by disease, is doing its office with facility, which for some time past has not been the case with me.

"6th. I have not bathed again, but I still feel the effects of the bath in my mouth. My appetite is now good, and I sleep with tranquillity, which I had not done of late. With all this, my pulse is quicker than usual, and I am sensible of some degree of languor. It is, however, to be observed, that the weather is very hot; the thermometer being, during the day, in the shade, from 92 to 96 degrees of Fahrenheit.

"In order to get a delicate test for acidity or alkalescency, I have been accustomed to rub the red petals of the *hibiscus rosa sinensis* on white paper, where they leave a blue-coloured stain. I observe when under the influence of the bath, the urine no longer turns this colour red, but destroys it altogether.

"*June* 6th. For a fortnight after giving up the bath, I was sensible of some of its effects about my mouth, and my pulse remained too quick. I am now remarkably well. My liver seems to be sound, and I have experienced a happier change than I ever did from mercury."

After this I used the nitro-muriatic bath in a variety of cases, and often with agreeable results. I now wrote another letter, giving an account of its powers: this letter was published in London, but it attracted very little attention, being tried by few individuals. When I came to London, and had it in my power to have the nitro-muriatic bath employed for various states of diseases, I was, as may be supposed, very anxious to know if it could be as commonly applicable in this climate as it is within the tropics. I have been much pleased to discover that it gives here the very same results that I formerly derived from it; nor are the maladies for which it may bring relief, less general in this country than in India. On the contrary, they seem to be still more abundant.

The acid that I have used of late is the nitro-muriatic; and it is formed by mixing together equal parts of the nitrous, or nitric acid, and muriatic acid. If these acids be in the state of concentration that they usually possess in the shops, and if the quantities be considerable, a great volume of gas is evolved on their coming into contact, which taints every part of a house, is extremely hurtful to the lungs, and disagreeable to the smell. To avoid all this inconvenience, I put a quantity of water, at least equal in bulk to both the acids, into a bottle, and I add the acids to it separately. This method does not only prevent the unpleasant odour, but it tends to retain the chlorine, on which its effects depend. It is well known that the nitro-muriatic acid acts very readily on the metals and earth; nothing therefore but glass, or extremely well-glazed vessels of porcelain, should be used to contain it. Wooden tubs for bathing answer very well, and they should always be made *as small as possible,* compatibly with their holding the body, or the limbs that we wish to expose to the bath. From their being small we save save acid, and are able to heat the bath with ease. In India I have often exposed the whole body below the head to this bath; but here I have been satisfied in general by keeping the legs and feet exposed to it. In order to warm the baths after the first time, I have commonly made a third or a fourth part of it be thrown away, and the loss replaced by boiling water, and a proportional quantity of acid. To save the expenditure of acid, I have occasionally warmed a portion of the bath in porcelain vessels placed near the fire, but I fear this may diminish its effects.

It is no easy matter to give directions with regard to the degree of acidity of the bath. I have commonly made it about as strong as very weak vinegar, trusting to the taste alone. The strength should be regulated by the degree of irritability of the patient's skin. I may say, that, although I like to know that it is strong enough to prick the skin a very little, after being exposed to it from fifteen to thirty minutes, yet I believe that even such an effect as this is unnecessary.

The time, too, of remaining in the bath, in order to produce the greatest effect, is a matter of doubt; I have kept the legs and feet exposed to it for half an hour or more; but with more delicate people not above one-half, or one-third of that time. I have repeated these baths daily, or even twice or thrice a-day.

I must now observe that the mere sponging the skin with nitro-muriatic acid, sufficiently diluted with water, gives rise to the very same effects with bathing, and is more easily applied. Fifteen or twenty minutes may be employed in the sponging, though I have found that a much less time does produce very material effects. When the bathing or sponging is carried to a considerable extent, and when the system is much under its influence, a sense of weakness comes on occasionally, some nervous irritation and restlessness are felt, a taste of metal (generally compared to that of copper) becomes sensible; a sense of pain occurs in some part of the palate or mouth, which is not permanent, but comes and soon goes off again. At length little specks or small ulcerations, extending no deeper than the cuticle, are seen on the interior surface of the mouth, and over the tongue, so that some degree of excoriation or rawness is at last produced. This is attended by a considerable discharge of saliva, with an increase of the feeling or lowness of depression. These effects resemble those of mercury, but they are not the same. The excoriation from the nitro-muriatic acid never reaches deeper than the cuticle: it never gives rise to fœtid ulceration of any kind; nor does it produce the least offensive smell of the breath nor in the mouth. The effects of it in this way are surprisingly fugitive. At one hour the discharge of saliva may be excessive; the next it will stop, and perhaps suddenly come on again. The excoriations in the mouth generally go away in a day or two, if the remedy be discontinued, and appear no more. While the mouth in this way is affected by the acid, the teeth partake of uneasiness; but I never saw this in a considerable degree, nor have I known any injury done to the teeth or their sockets. These lastmentioned mentioned effects are seldom met with to the extent that I have described, and need not be excited unless some peculiar circumstances require an unusual power, such as the symptoms of syphilis. I have lately added more and more of the muriatic acid in proportion to the nitric, and the effects have proportionally increased. I now make use of equal parts of the acids. Would not the greatest power of the remedy be obtained by those proportions that produce the greatest quantity of chlorine; for from that element I believe all its effects arise? I am yet ignorant what effects would be produced by the muriatic acid alone.*

The nitro-muriatic acid appears in a particular manner to affect the glands, and to alter their secretions; and on this power a great part of its value in derangements of the liver seems to depend. It sometimes very suddenly increases the secretion of bile; and this effect may be kept up for a length of time. It increases the perspiration, and often to a great extent. Whether the nitro-muriatic acid be applied to the inner surface of the stomach, or to the external surface of the body, the effect is the same in kind, though not in degree. As a very general rule for its employment, it may be observed, that, whenever the mercurial preparations are indicated, the nitro-muriatic acid will be found useful, with this difference, that, in cases where mercury is highly injurious from delicacy or peculiarity of constitution, or from other causes, the nitro-muriatic acid may be employed with safety and advantage. On the other hand, I should not at present recommend it in acute diseases, with the exception of some kinds of fever, and of hepatitis of every character. Where the pulse is quick, and where there is an inflammatory tendency, I think it would be injurious.

I was first led to the use of the nitro-muriatic acid from an attention to the diseases of the liver. The derangements of that organ, and of its secretion, open a vast field for inquiry, which has been but imperfectly explored. I shall, as an example of the application of the nitro-muriatic acid, say a few words on this important subject.

### *Acute and Chronic Hepatitis.*

With regard to acute hepatitis, if pure and unmixed, the propriety of employing the nitro-muriatic acid might admit of a doubt; but the same observation may be applied to mercury. I think, however, that I never met with a case of acute hepatitis that did not partake of the chronic affection, either at its commencement or soon afterwards. I know, from experience, that, within the tropics, where I lived so long, a proper use of mercury is never to be neglected in either affection of that organ. I have not trusted to the acid where I thought the risk of abscess considerable; but without delay have employed mercurials, and every other means in my

* Since I wrote the above, some of my friends, as well as myself, have used, for sponging the skin, chlorine dissolved in water, and with the same effects exactly as arise from the union of the acids.

power, to prevent, if possible, a termination so lamentable. I should now not hesitate to add this new power to the other means; and have no doubt, if really insufficient of itself, it would greatly aid us in affording security and relief.

Chronic hepatitis is a far more common disease than the acute; but it may be considered as always partaking of the nature of both. One portion of the same liver is often insensible, enlarged, and inactive, while another part of it is suffering from all the symptoms of acute hepatitis, and going on to the formation of pus. It is this mixed disease that we meet with so generally in India, as well as in this country; and it is this state of the liver which gives rise to so great a variety of anomalous symptoms.

For this chronic affection, it appears to me, that the nitro-muriatic acid applied to the skin is the most effectual and the safest remedy. A few hours, or even a single hour, will sometimes bring relief; but it is necessary to continue the remedy till the system be sufficiently affected by it, and to repeat it occasionally *till the patient has recovered his usual degree of strength.* This is a rule, in affections of the liver, of the utmost importance. A state of weakness, however produced, is the great remote cause of those chronic affections; so that we may remove the disease, but, till the strength and vigour of the circulation be restored, we have no security against a return of it.

This affection of the liver produces a vast variety of diseases, to which various names have been assigned. To describe them well would require much time, and occupy many volumes. The process of digestion has an intimate connection with the bile: if this be depraved, the stomach and intestinal canal partake immediately of the disorder. The brain, which seems to be the source of feeling and motion, is connected, by means of numerous nerves, with those abdominal viscera by which so many of the functions of life are carried on. A diseased state of the bile has a wonderful influence on the whole nervous system: it gives rise to pain and giddiness of the head; a great dislike to motion, and a sense of weakness, rather than actual weakness; cramps come on in the legs while asleep; the soles of the feet are tender and painful, and at times the sick rather drag them than raise them when they walk. A most able and intelligent friend, who was lately relieved very suddenly by the nitro-muriatic acid bath, from a state of long-continued nervous irritation, is of opinion, that all the misery he had suffered for years arose from a depraved state of the biliary secretion. His observations on himself are curious, and very important. He had long taken notice that the bile, though at times ample in quantity, was insoluble in water; and that the fæces had lost entirely the fæcal odour. The solubility in water, with this peculiar odour, gradually returned from the use of the remedy, while at the same time the irritability of the nervous system was suddenly corrected. But I think there is sufficient evidence that this acid, in some cases, acts directly on the nervous system; for in some people I have seen it very suddenly produce a sense of composure, of quiet, and of hap-

 pines, piness, and for days together they have been sensible of a degree of an agreeable intoxication.

There are other symptoms, though less obscure, perhaps, in their origin, that are often connected with the chronic affection of the liver. The blood cannot pass through it with the facility necessary to health, nor is it possible to relieve it effectually from such a state, but by giving solubility to the bilious matter. It then passes off abundantly through the proper channels into the duodenum. If, from obstruction or enlargement, the blood be prevented from circulating with ease in the liver, a general disorder of the whole frame becomes apparent. The feet and ankles swell, and a fulness in the head comes on, with head-ache and giddiness, and a train of unhappy feelings. In this country it is a common practice to have recourse to the abstraction of blood by cupping, or by the lancet, in order to alleviate such symptoms. The first effect of this practice is, no doubt, occasionally to give relief to the head; but this is only temporary. An equal quantity of blood is again accumulated; a repetition of blood-letting is required; the state of weakness continues to increase; and the patient falls a victim at last to an injurious practice, derived from a theory altogether erroneous. It is not by letting off a part of the blood that we can do any good; for it is neither too abundant in quantity, nor bad in quality. The fulness of the head, as well as of the feet, does really arise from the remora to the blood in another portion of its circuit; and in both extremities of the body it is produced by one and the same cause.

I need hardly say that melancholy and despondency of mind are often connected with a peculiar state of the bile, for this has been observed in all ages. This state of mind I have often seen removed by a proper use of nitro-muriatic bath; and people of both sexes have assured me, that they think it had preserved them from the crime of suicide, to which, during the horrors of their feelings, they had an alarming tendency.

That state of the bile already mentioned, in which it seems to be deficient in quantity, and probably at the same time unhealthy in its nature, is very common. Of all hepatic affections, I think this attended with the most pain and distress of the bowels. This disorder of the biliary system frequently gives rise to a flux, which I have known to go on for many months, and even for years. I have generally seen a deficiency of bile without a tendency to flux, and often even accompanied by a constipated state of the primæ viæ. Such a condition of the liver and bile does frequently give rise to most uneasy derangements of the stomach; a tendency to acidity or heartburn; little uccerations over the surface of the mouth and œsophagus, and perhaps extending downwards through the whole track of the intestinal canal. This aphthous disease is very distressing and dangerous, though I have been very successful in curing it by the nitro-muriatic acid. I know of no other remedy for this affection of the stomach and intestines, as the common means of cure seem to me to be very far from sufficient.

I may say with truth, that in such a condition of the liver and bile, bile, all remedies that stimulate or excite the circulation are *injurious.* Among such I may reckon wine, spirits, bark, bitters, and steel. With such agents opium has been classed; but many of its effects are peculiar to itself, and a proper application of it in such complaints is often of the utmost importance and utility. In almost every state and stage of diseased liver, opium may be given to many with benefit. Even in acute hepatitis it answers a salutary purpose when combined with calomel, or with the quicksilver pill: for, unlike what occurs in other inflammatory affections, it seems in those of the liver to be unattended by almost any ill effect. In chronic hepatitis, it alone is able to calm the irritability and unhappy feelings, and to allow time for the application of the means of cure. It seems, indeed, by its sedative power, to have a beneficial influence on the liver, and perhaps to do something more than produce a temporary calm. Opium, however, should never be given in chronic hepatitis, without great attention to its effect in diminishing the propulsive motions of the primæ viæ. I have been accustomed to give an opiate at bed-time, together with some laxative substance, such as the sulphate of magnesia, to correct its constipating effect.

It is proper to observe, that bilious people, and especially those who have been subject to intermittents, after finding the utmost relief from mercury, or the nitro-muriatic acid, and at the moment when we think they are advancing fast towards a perfect recovery, are apt, all at once, to say they are unwell, to lose their appetite, to become a little feverish, and to complain of their head. If these symptoms be neglected, the patient soon gets regular attacks of a quotidian fever, beginning with a cold fit, and followed by the ordinary stages of that disease. Such is the connection which a great flow of bile, however excited, appears to have with fever of the intermittent kind.*

Besides this fever, which is of the true intermittent kind, there is very generally a troublesome symptomatic fever, that plagues bilious people. It steals on imperceptibly, and, when once begun, gives rise, with little other inconvenience, to a state of most obstinate sleeplessness. This too may be obviated by an opiate, given before the hour of its accession; but it can be cured only by correcting the cause of it. Opiates, with bilious people, are very apt to occasion

* Although I have not seen this fever under a violent form, yet it is depressive and inconvenient. It is easily stopped by the Peruvian bark, taken daily in decoction, before the hour of the fit, and repeated at the same time for a few succeeding days. A common wine glass full of it, every two hours, and repeated for three or four times before the fever is expected, I have always found to be sufficient for curing it. With some people, I judge it prudent to give the bark decoction during the whole of the time they use the acid. It is often very useful to add a few drops of laudanum to each glassful of the bark decoction.

great itching of the skin, by delaying the passage of the bile through the bowels.

From a diseased state of the bile, the memory is often affected, and a degree of stupidity, and even of idiotcy, comes on. From this cause, too, the hair at times grows harsh and hard to the feeling; and I have seen it, like the skin, become soft and flexible from the use of the nitro-muriatic acid. In both cases I imagine that the effect arises from the long-suppressed matter of perspiration being abundantly restored. To all these I may add another inconvenient complaint from a bilious state—a frequent desire to pass the urine. Whether this irritation arise from a diseased sensibility of the whole nervous system, or from a morbid alteration in the urinary fluid, I do not know.

In all biliary derangements the rule is never to be forgotten, and I repeat it again, that *there is no security against a relapse till the health and strength are fully restored; and that, till then, some repetitions of the remedy are necessary.*

The good effects of the nitro-muriatic practice can never be appreciated until it has been discontinued for several weeks, or rather months. During the use of the bathing or sponging, the pulse is often very quick, and patients grow thinner, even when they feel better. At times, too, they often complain of more than usual uneasiness in the liver, or in the region of it; they often lose their flesh, and look very yellow. The remedy seems to alter and agitate that organ. The flow of bile, when once excited, goes on for a number of days; and not, with some people, without inconvenience. If it do not affect the bowels as a laxative, it is highly necessary at this time to employ some substance that has this power, such as the sulphate of magnesia, senna, or aloes. In time, however, it is commonly found that the health, the strength, and the colour of the skin are much improved.

It is no small advantage of this practice, that we can apply the power in as high a degree as the strongest can bear, or in quantities so minute, that the most sensitive and nervous being can hardly be injured by it. I have immersed many to the chin in this bath, and I have been afraid, in other cases, to wet more than a single hand with the acid. The length of time, too, that the acid remains in contact with the skin may be infinitely varied. We have thus a power extremely divisible, and applicable to almost every degree of resistance or sensibility.

When the acid produces very considerable effects, it is right, after a few days, to stop its use for a week or two; as, if used long, it gives rise to unnecessary uneasiness from bilious discharges or bilious feelings. I have said that drinking the nitro-muriatic acid has the very same effects with its external use. When taken in this way, it should be very much diluted with water. Indeed it should taste but very slightly acid, and be drank in small portions at a time. I need hardly say, that it is very necessary to take care that, even in this weak state, it be not allowed to touch the teeth. The mouth should be immediately washed after swallowing it, and every precaution precaution employed that is used with the mineral acids, to prevent injury from its external action. This way of using the nitro-muriatic acid is often very convenient and salutary, and in many cases may deserve a preference to any other. I think it is particularly applicable to some states of indigestion, and when we wish to produce effects gradually and insensibly.

[Some inquiries follow concerning the *modus operandi*, of which, however, Dr. Scott candidly confesses his total ignorance; but, that the power depends on the chlorine, as Sir H. Davy terms it,* he has no doubt. This is further explained in a supplementary note.]

P. S.—Since I wrote the preceding paper, several of my friends have become convinced with me, that the very same effects arise from a diluted solution of chlorine in water, as are produced by the nitro-muriatic acid. Our late experience puts an end to all doubt, if any could have existed, that chlorine alone is the source of the material effects. We have sponged the skin with a solution of chlorine in water, and in many cases, have obtained the same results as arise from a similar application of the nitro-muriatic acid. The solution of chlorine to which I allude, is water through which the oxi-muriatic acid gas has been made to pass, until it could retain no more of it. This mode of applying chlorine has the advantage of not irritating the most sensible skins so much as the diluted nitro-muriatic acid might do; but even this solution I have diluted with about four times its bulk of water, before I applied it.

It is remarkable that the aqueous solution of chlorine, procured by mixing the acids together, is far less offensive than its solution got by the common means of passing the gas through water. Some degree of affinity seems in the first case still to subsist between it and the other elements of the acids, by which its sensible qualities are diminished to a certain extent. We are under great obligation to Sir Humphry Davy, for the light he has lately thrown on this subject, by which the effects of the *aqua regia* of the chemists are clearly accounted for.

I have of late received from different quarters, and from competent judges, a confirmation of the opinions I had expressed of the effects of chlorine in derangements of the liver, and in syphilitic affections. As those opinions were derived from experience alone, I cannot but think that a future day will confirm them.

I am told that some others have been less successful than myself or my friends with this remedy, and that by the application of chlorine to the skin, they have been unable to produce a sensible effect of any kind. I can make no reply to such opinions, as I do not know how the trials on which they rest have been conducted; but I must affirm, that I have sooner or later been able to produce very distinct effects in almost every case in which I have employed it.

* For Sir H. Davy's Theory of Chlorine, see our Journal, vol. xxxv. p. 111.

Time will decide between us, but on one side of the question I need not say, there is a want of right observation.

If it be considered that the most active of all the mercurial preparations in use are calomel, (submuriate of mercury,) and corrosive sublimate, (oxymuriate of mercury,) we may ascribe this great activity to the chlorine of the composition. Why the sanative powers of the mercurial preparations were supposed to arise from the metal alone, I cannot conceive. In like manner the chemists for a long time neglected the water that might be mixed with the materials of their experiments, the elements of which water gave rise to effects that misled them in all their reasonings. I am now nearly, I think, in a condition to shew what effects in the system arise from mercury as a metal, and what effects are derived from the other element of the mercurial preparations, whether this metal in them be combined with oxygen or with chlorine.

